# Supplementary material for: Passive electroculture using copper rods does not improve yield in home container vegetable gardening
Source: PLoS One. 2025 Aug 7;20(8):e0329615. doi: 10.1371/journal.pone.0329615 (PMC12331079; doi:10.1371/journal.pone.0329615)

PLOS ONE

Passive electroculture using copper rods does not improve vegetable yield.

--Manuscript Draft--

**Manuscript Number:**	PONE-D-25-18809
**Article Type:**	Research Article
**Full Title:**	Passive electroculture using copper rods does not improve vegetable yield.
**Short Title:**	Copper Rods Do Not Improve Plant Performance
**Corresponding Author:**	Nathan Lemoine<br>Marquette University<br>UNITED STATES OF AMERICA
**Keywords:**	Photosynthesis; electroculture; electricity; agriculture; gardening; aboveground biomass; belowground biomass
**Abstract:**	Electroculture, or the practice of applying electric fields or current to plants, has been explored for nearly three hundred years. Recently, home gardeners on social media have adopted the term electroculture to describe inserting copper-wrapped dowels into root soil as a cost-effective means of improving crop yield in small garden spaces. Given the renewed interest, big box stores have begun stocking copper-wrapped dowels as a means of improving plant growth in urban container gardens, yet whether such passive electroculture is sufficiently beneficial to plant growth to justify the cost and materials, particularly of copper, remains debated. It is likely that copper rods produce too little voltage to affect plant physiology. In this study, we tested the hypotheses that 1) inserting copper-wrapped dowel rods into the soil will not improve plant growth, photosynthesis, or yield, and 2) if copper-wrapped dowel rods improve plant growth, it is due to copper fertilization rather than electrical conductance. We tested these hypotheses on two leafy green vegetables, mustard greens and kale, and two root vegetables, beets and turnips, to determine if plant life history was an important factor in the efficacy of passive electroculture. We found no consistent evidence that passive electroculture is beneficial to crop growth or yield in container gardens. Although we documented statistically significant effects of buried copper on the above- and belowground biomass of turnips, it is unlikely that improved turnip yield was due to copper fertilization because plants grown with exposed copper rods did not show the same effect. While crop production could potentially be enhanced by the application of electrical fields, the voltages required exceed what is produced by copper-wrapped wooden dowels. We therefore suggest that both the production and purchase of such products would waste both financial and natural resources.
**Order of Authors:**	Mya Chier
	Aidan Oakey
	Michelle L Budny
	Nathan Lemoine
**Additional Information:**	
**Question**	**Response**
**Financial Disclosure**<br><br>Enter a financial disclosure statement that describes the sources of funding for the work included in this submission. Review the submission guidelines for detailed requirements. View published research articles from *PLOS ONE* for specific examples.	Yes

Powered by Editorial Manager® and ProduXion Manager® from Aries Systems Corporation

This statement is required for submission and **will appear in the published article** if the submission is accepted. Please make sure it is accurate.<br><br>**Funded studies**<br>Enter a statement with the following details:<br>• Initials of the authors who received each award<br>• Grant numbers awarded to each author<br>• The full name of each funder<br>• URL of each funder website<br>• Did the sponsors or funders play any role in the study design, data collection and analysis, decision to publish, or preparation of the manuscript?<br><br>Did you receive funding for this work?	
Please add funding details.<br>as follow-up to "**Financial Disclosure**<br><br>Enter a financial disclosure statement that describes the sources of funding for the work included in this submission. Review the submission guidelines for detailed requirements. View published research articles from *PLOS ONE* for specific examples.<br><br>This statement is required for submission and **will appear in the published article** if the submission is accepted. Please make sure it is accurate.<br><br>**Funded studies**<br>Enter a statement with the following details:<br>• Initials of the authors who received each award<br>• Grant numbers awarded to each author<br>• The full name of each funder<br>• URL of each funder website<br>• Did the sponsors or funders play any role in the study design, data collection and analysis, decision to publish, or preparation of the manuscript?	NSF DEB 1941390

Did you receive funding for this work?"	
Please select the country of your main research funder (please select carefully as in some cases this is used in fee calculation).<br>as follow-up to "**Financial Disclosure**<br><br>Enter a financial disclosure statement that describes the sources of funding for the work included in this submission. Review the submission guidelines for detailed requirements. View published research articles from *PLOS ONE* for specific examples.<br><br>This statement is required for submission and **will appear in the published article** if the submission is accepted. Please make sure it is accurate.<br><br>**Funded studies**<br>Enter a statement with the following details:<br>• Initials of the authors who received each award<br>• Grant numbers awarded to each author<br>• The full name of each funder<br>• URL of each funder website<br>• Did the sponsors or funders play any role in the study design, data collection and analysis, decision to publish, or preparation of the manuscript?<br><br>Did you receive funding for this work?"	UNITED STATES - US
**Competing Interests**<br><br>Use the instructions below to enter a competing interest statement for this submission. On behalf of all authors, disclose any competing interests that could be perceived to bias this work—acknowledging all financial support and any other relevant financial or non-financial competing interests.<br><br>This statement is required for submission and **will appear in the published article** if	The authors have declared that no competing interests exist.

the submission is accepted. Please make sure it is accurate and that any funding sources listed in your Funding Information later in the submission form are also declared in your Financial Disclosure statement.<br><br>View published research articles from *PLOS ONE* for specific examples.<br><br>**NO authors have competing interests**<br><br>Enter: *The authors have declared that no competing interests exist.*<br><br>**Authors with competing interests**<br><br>Enter competing interest details beginning with this statement:<br><br>*I have read the journal's policy and the authors of this manuscript have the following competing interests: [insert competing interests here]*<br><br>* typeset	
**Ethics Statement**<br><br>Enter an ethics statement for this submission. This statement is required if the study involved:<br><br>• Human participants<br>• Human specimens or tissue<br>• Vertebrate animals or cephalopods<br>• Vertebrate embryos or tissues<br>• Field research<br><br>Write "N/A" if the submission does not require an ethics statement.<br><br>General guidance is provided below. Consult the submission guidelines for detailed instructions. **Make sure that all information entered here is included in the Methods section of the manuscript.**	N/A

**Format for specific study types**<br><br>**Human Subject Research (involving human participants and/or tissue)**<br>• Give the name of the institutional review board or ethics committee that approved the study<br>• Include the approval number and/or a statement indicating approval of this research<br>• Indicate the form of consent obtained (written/oral) or the reason that consent was not obtained (e.g. the data were analyzed anonymously)<br><br>**Animal Research (involving vertebrate animals, embryos or tissues)**<br>• Provide the name of the Institutional Animal Care and Use Committee (IACUC) or other relevant ethics board that reviewed the study protocol, and indicate whether they approved this research or granted a formal waiver of ethical approval<br>• Include an approval number if one was obtained<br>• If the study involved *non-human primates*, add *additional details* about animal welfare and steps taken to ameliorate suffering<br>• If anesthesia, euthanasia, or any kind of animal sacrifice is part of the study, include briefly which substances and/or methods were applied<br><br>**Field Research**<br><br>Include the following details if this study involves the collection of plant, animal, or other materials from a natural setting:<br>• Field permit number<br>• Name of the institution or relevant body that granted permission	
**Data Availability**<br><br>Authors are required to make all data underlying the findings described fully available, without restriction, and from the time of publication. PLOS allows rare exceptions to address legal and ethical concerns. See the PLOS Data Policy and FAQ for detailed information.	Yes - all data are fully available without restriction

A Data Availability Statement describing where the data can be found is required at submission. Your answers to this question constitute the Data Availability Statement and **will be published in the article**, if accepted.<br><br>**Important:** Stating 'data available on request from the author' is not sufficient. If your data are only available upon request, select 'No' for the first question and explain your exceptional situation in the text box.<br><br>Do the authors confirm that all data underlying the findings described in their manuscript are fully available without restriction?	
**Describe where the data may be found in full sentences. If you are copying our sample text, replace any instances of XXX with the appropriate details.**<br><br>• If the data are **held or will be held in a public repository**, include URLs, accession numbers or DOIs. If this information will only be available after acceptance, indicate this by ticking the box below. For example: *All XXX files are available from the XXX database (accession number(s) XXX, XXX.).*<br>• If the data are all contained **within the manuscript and/or Supporting Information files**, enter the following: *All relevant data are within the manuscript and its Supporting Information files.*<br>• If neither of these applies but you are able to provide **details of access elsewhere**, with or without limitations, please do so. For example:<br><br>*Data cannot be shared publicly because of [XXX]. Data are available from the XXX Institutional Data Access / Ethics Committee (contact via XXX) for researchers who meet the criteria for access to confidential data.*<br><br>*The data underlying the results presented in the study are available from (include the name of the third party*	10.6084/m9.figshare.28752290

and contact information or URL).<br>• This text is appropriate if the data are owned by a third party and authors do not have permission to share the data.<br><br>* typeset	
Additional data availability information:	

Manuscript

Click here to access/download;Manuscript;Electroculture_2025-04-08_NL.doc

~~**Passive Electroculture Using Copper Rods Does Not Improve Vegetable Yield**~~

Mya Chier[1], Aidan Oakey[1], Michelle L Budny[1], Nathan P Lemoine[1,2]

[1]Department of Biological Sciences, Marquette University, Milwaukee WI USA

[2]Department of Zoology, Milwaukee Public Museum, Milwaukee WI USA

corresponding author: nathan.lemoine@marquette.edu

Abstract

Electroculture, or the practice of applying electric fields or current to plants, has been explored for nearly three hundred years. Recently, home gardeners on social media have adopted the term electroculture to describe inserting copper-wrapped dowels into root soil as a cost-effective means of improving crop yield in small garden spaces. Given the renewed interest, big box stores have begun stocking copper-wrapped dowels as a means of improving plant growth in urban container gardens, yet whether such passive electroculture is sufficiently beneficial to plant growth to justify the cost and materials, particularly of copper, remains debated. It is likely that copper rods produce too little voltage to affect plant physiology. In this study, we tested the hypotheses that 1) inserting copper-wrapped dowel rods into the soil will not improve plant growth, photosynthesis, or yield, and 2) if copper-wrapped dowel rods improve plant growth, it is due to copper fertilization rather than electrical conductance. We tested these hypotheses on two leafy green vegetables, mustard greens and kale, and two root vegetables, beets and turnips, to determine if plant life history was an important factor in the efficacy of passive electroculture. We found no consistent evidence that passive electroculture is beneficial to crop growth or yield in container gardens. Although we documented statistically significant effects of buried copper on the above- and belowground biomass of turnips, it is unlikely that improved turnip yield was due to copper fertilization because plants grown with exposed copper rods did not show the same effect. While crop production could potentially be enhanced by the application of electrical fields, the voltages required exceed what is produced by copper-wrapped wooden dowels. We therefore suggest that both the production and purchase of such products would waste both financial and natural resources.

Introduction

Electroculture, or the practice of applying electric fields or current to plants [1], has been explored for nearly three hundred years. In the 18th century, Jean-Antoine Nollet reported that applying electrical current improved plant growth [2]. In the late 19th century, Selim Lemström reported that electrical stimulus improved the growth of potatoes, celery, and carrots by 40 – 70% over a two month period [3]. Throughout the early- and mid-20th century, electroculture received sporadic attention as a potential means of improving agricultural yield [4–7], although the practice has never been widely adopted in commercial settings. Recently, home gardeners on social media have adopted the term electroculture to describe inserting copper-wrapped dowels into root soil as a cost-effective means of improving crop yield in small garden spaces [8]. Given the renewed interest, big box stores have begun stocking copper-wrapped dowels as a means of improving plant growth in urban container gardens, yet whether such passive electroculture is sufficiently beneficial to plant growth to justify the cost and materials, particularly of copper, remains debated.

Electrical stimulation could, theoretically, improve photosynthesis and thereby enhance crop production by mimicking electrical signaling pathways. For example, electrical signaling appears to be among the first rapid signals to stress, which ultimately helps maintain net CO_2 uptake [9]. During temperature stress, electrical signals help maintain stomatal CO_2 uptake, potentially preventing stomatal closure caused by dropping foliar water potentials [10]. Electrical signals also propagate from the roots up to the leaves, serving as a potential signaling mechanism to increase photosynthetic activity in the presence of increased nutrient supply [11]. These lines of evidence suggest that an applied electrical current might potentially aid crop production by maintaining CO_2 uptake, particularly during stressful events. However, electrical stimulus can

also serve to depress photosynthesis as a response to foliar damage, similar to the effect of mechanical wounding on leaves [12,13]. In such negative feedbacks, electrical stimuli might serve to repress photosynthesis and crop yield. Indeed, strong electrical fields and currents have a multitude of effects, such as increased yield and germination rate, decreased yield, increased chemical defense production (similar to defense induction by wounding), or decreased or increased germination times [14] . The effect of electrical stimulus appears to depend on both the plant species and the type and magnitude of electrical current applied [14], and whether copper dowels provide enough electricity to promote crop growth remains untested.

It is also possible that plant physiology and performance can be altered by ~~directly by the~~ copper coil ~~inserted~~ into the soil. At high concentrations, copper becomes photo-inhibitory and forms the base metal of many herbicides. Experiments have repeatedly demonstrated that excess copper can inhibit photosynthesis [19,20]. Interestingly, photosynthesis appears to be inhibited during carbon fixation, rather than via stomatal limitation [20]. At low concentrations, however, copper amendments can improve yield. Copper fertilization increased wheat photosynthesis and yield by over 100% in copper-limited soils of Canada and India, and also stimulated photosynthesis of hydroponically grown rice [15–17]. Small amount of copper contained in fungicide applications also resulted in a temporary increase in CO_2 assimilation of hops [18]. Yet no controlled study exists to determine whether the benefits of passive electroculture are due to copper fertilization rather than electrical stimulation of plants.

In this study, we tested two hypotheses:

H1: Inserting copper-wrapped dowel rods into the soil will not improve plant growth, photosynthesis, or yield. Although electrical stimulation can improve plant performance, it is unlikely that a copper dowel passively transmits enough electricity into the soil aid plant health. We therefore predicted that CO_2 assimilation, growth rate, and biomass should be similar between plants grown with and without copper-wrapped dowels.

H2: If copper-wrapped dowel rods improve plant growth, it is due to copper fertilization rather than electrical conductance. If true, we predicted that burying copper-wrapped dowel rods severed at the soil surface would enhance plant performance similarly to copper-wrapped dowels that extend above the soil surface.

We tested these hypotheses on two leafy green vegetables and two root vegetables to determine if plant life history was an important factor in the efficacy of passive electroculture.

Methods

Plant germination

We selected four common garden species for our study: two leafy green varieties, mustard greens (*Brassica* spp.) and white Russian kale (*Brassica* spp.), and two root vegetables, turnips (*Brassica* spp.) and beets (*Beta* spp.). Seeds germinated in an environmental growth chamber (Conviron GEN1000; Winnipeg, Canada) set to 24 °C, 85% relative humidity, and 15:9 hour photoperiod. After four weeks we transplanted seedlings into individual one-gallon pots with garden potting soil and moved the pots to a lab bench under LED grow lights. Ambient room temperature stayed near 25 °C. We measured soil moisture weekly with a TDS Field Scout soil moisture meter and watered plants to maintain 20-30% soil moisture.

Copper stimulation of plant growth

We tested the effects of copper electroculture by randomly assigning individual plants to one of three treatments: a control group with no added copper, exposed copper wire, and buried copper wire (*n*=10 plants per treatment, per species). The exposed copper wire treatment consisted of a 40 cm dowel rod coiled with copper wire and inserted 10 cm into the soil. To control for potential copper fertilization effects, we buried 10 cm dowel rods coiled with copper wire into the pots completely covered by the soil. Each week we measured plant height from the soil surface to the highest point on the stem. After eight weeks, all aboveground biomass was clipped and dried to a constant weight, and belowground biomass of beets and turnips was harvested, dried and weighed. We did not measure belowground biomass for mustard greens and kale since their roots were too fine to separate from the soil without substantial root damage or loss.

Plant physiology measurements

We measured leaf greenness weekly using a non-destructive SPAD meter (Konica-Minolta) as a proxy for chlorophyll content, which requires destructive sampling. Net CO_2 assimilation (A_{net}) and stomatal conductance to water vapor (g_{sw}) were measured using an LI-6800 portable photosynthesis analyzer (Li-COR, Lincoln, Nebraska, USA). We measured A_{net} and g_{sw} on young fully expanded leaves with no signs of damage under the following conditions: flow rate = 400 μmol s^{-1}, CO_2 reference = 420 μmol mol^{-1}, relative humidity = 50%, light = 1500 μmol m^{-2} s^{-1}, fan speed = 10,000 rpm, leaf temperature = ambient conditions. We enabled dynamic equations on the LI-6800 to calculate A_{net} [21]. Leaves were allowed to acclimate until A_{net} was stable (maximum of 2 minutes) before logging data.

Statistical Analyses

We tested treatment effects of copper wire on A_{net}, g_{sw}, height, and SPAD using a repeated measures ANOVA. We compared the likelihood ratio of the basic model (week) to the treatment model (week + treatment), and the treatment model to the interaction model (week + treatment + week:treatment). If a likelihood ratio was significant (p<0.05), we conducted a Tukey pairwise *post-hoc* test to determine which groups differed. To reduce the number of post-hoc tests and therefore the severity of the p-value corrections, we only compared treatments within a given week and not across weeks. We assessed water use efficiency (WUE) by log-transforming A_{net} and g_{sw} and performing an ANCOVA to test the slopes (i.e. CO_2 uptake / H_2O loss) by treatment.

To find the treatment effect of copper on aboveground biomass of each species, and belowground biomass of turnips and beets, we performed ANOVAs of each treatment. If the ANOVA was significant, we used a Tukey pairwise test to find which treatments had a significant interaction.

Results

Physiology

Neither exposed nor buried copper affected A_{net} of kale, mustard greens, or turnips, while beets had a significant ~~an~~ interaction between copper treatment and week (Fig. 1, Table 1). In week 6, beet plants with exposed copper had lower A_{net} than both control plants (p < 0.001) and plants with buried copper (p < 0.001), and control plants did not differ from plants with buried copper (p = 0.63, Fig. 1C). However in week 8, plants with buried copper had lower A_{net} than the control plants (p = 0.02) and plants with exposed copper (p = 0.04). We found no effect of copper treatment on g_{sw} for mustard greens, beets, or turnips. Kale, on the other hand, did show a treatment effect but not for the interaction with week (Table 1). Kale plants with buried copper

had 13% higher g_{sw} than the control plants (p = 0.02) and 18% higher g_{sw} than plants with exposed copper (p < 0.01) over the course of the experiment (Table 1, Fig. 2).

We found no interaction of copper treatment and WUE for kale, mustard greens, or beets (Fig. 3, Table 2). Initially we did find an interaction of WUE in turnips. The slope of the control plants was shallower than the slope for exposed and buried, suggesting that control plants had lower WUE than either copper treatment. However, the shallow slope was driven by the inclusion of one outlier that, when excluded, removed the effect of copper treatment on WUE for control plants (Fig. 3, Table 2).

Height and SPAD

Neither treatment nor the interaction of treatment and week was significant for height or SPAD for any species (Table 1).

Biomass

Copper treatments affected aboveground biomass production of the root vegetables but not the leafy greens (Fig. 4). Among treatments, we found no differences in aboveground biomass of mustard greens (p=0.09) nor kale (p=0.83). Beet and turnip plants with buried copper both produced more aboveground biomass than their control plants (both p < 0.01). Compared to exposed copper, buried copper marginally increased beet aboveground biomass (p = 0.054), but did not affect turnip aboveground biomass (p = 0.08). Neither beet nor turnip aboveground biomass differed between exposed copper and control treatments (p = 0.42 and 0.27, respectively). However, control plant biomass was lower than the mean of combined copper treatments (p = 0.01) for both species.

We did not find any effect of copper on belowground biomass production except for turnips with buried copper (Fig. 5). Turnip plants with buried copper had greater belowground biomass that the control (p = 0.01) and plants with exposed copper (p = 0.03), and control plants did not differ from exposed copper (0.83) or from the mean of combined copper treatments (0.06). Beet belowground biomass did not differ among treatments (p = 0.44).

Discussion

In this study, we tested the hypotheses that copper-wrapped dowel rods would not improve plant physiology, but if they did then the effect would be attributed to copper fertilization rather than electrical stimulation. We found no consistent evidence that passive electroculture is beneficial to crop growth or yield in container gardens. Although we documented statistically significant effects of buried copper on the above- and belowground biomass of turnips, it is unlikely that improved turnip yield was due to copper fertilization because plants grown with exposed copper rods did not show the same effect.

Several centuries of research have demonstrated that crop growth and yield are improved by electrical stimulation [2,5,6]. However, electrical voltages range between 6 – 1200 V, with most tests using over 200 V to assess the effectiveness of electroculture [14]. In fact, one study found that application of a 40,000 V electrical field improved wheat yields [22]. The lowest tested voltage reported in [14], 6 V, increased both above and belowground biomass of tomato plants [23]. While the studies confirm that electricity can be harnessed to improve plant growth, it is unlikely that passive electrical transport of copper rods provides enough voltage ~~affect~~ plants. Electrical transmittance to the soil requires a voltage differential between the soil and the

atmosphere. The commonly accepted differential is 100 V per meter, such that the extreme voltage differential between the soil and the top of a 40 cm copper rod is only 4 V. We measured electrical transmission directly using an oscilloscope and found that copper rods placed randomly around the room transmit only 2 millivolts into the soil. When placed within 4 cm of a fluorescent light, transmittance increased to a maximum of 20 millivolts. Thus, copper rods do transmit electricity into the soil, but at insignificant voltages that are unlikely to have any impact on plant physiology or performance.

We also hypothesized that the insertion of copper rods into the soil might provide fertilization benefit. At low concentrations, copper fertilization can improve crop yield in copper-limited soils [16–18]. However, many potting soils for home container use contain copper sulfate as an anti-bacterial and anti-fungal agent, likely providing plants with adequate copper. Naturally, soil copper concentrations in both the US [24] and Europe [25] are sufficient to alleviate copper limitation, with potential exceptions in the southeast US, western Michigan, and eastern Europe and Scandanavia. Indeed, we did find that buried copper rods increased aboveground biomass of both root vegetables, beets and turnips, by ~2.5 g (Fig. 4), and also increased belowground turnip yield by 1 g (Fig. 5). Yet we cannot conclude that these effects are due to copper fertilization because the exposed copper rods, with a similar amount of copper buried in the soil, did not increase either the above or belowground biomass of root vegetables (Figs. 4,5).

In summary, we have conducted a controlled experiment on multiple crops, including leafy greens and root vegetables, and found no evidence that passive electroculture can improve plant growth, photosynthesis, or yield. While crop production could potentially be enhanced by

the application of electrical fields, the voltages required exceed what is produced by copper-wrapped wooden dowels. We therefore suggest that both the production and purchase of such products would waste both financial and natural resources.

Acknowledgements

We would like to thank Tom Dunk for his electrical expertise. This work was partially funded by NSF DEB 1941390.

References

1. Pohl HA. Electroculture. Journal of Biological Physics. 1977;5: 1–33.
2. Nollet M l'Abbe. Recherches sur les Causes Particulieres des Phenomenes Electriques et sure les Effets Nuisibles ou Avantageux Qu'on Peut en Attendre. H L Guerin & L F Delatour; 1747.
3. Lemstrom S. Electricity in Agriculture and Horticulture. London: George Tucker; 1904.
4. Briggs LJ, Heald RH, Flint LH. Electroculture. United States Department of Agriculture; 1926. Report No.: 1379.
5. Jorgensen I, Stiles W. The Electroculture of Crops. Science Progress. 12: 609–621.
6. Christianto V, Smarandache F. A Review on Electroculture, Magneticulture and Laserculture to Boost Plant Growth. Infinite Study; 2021.
7. Pohl HA, Todd GW. Electroculture for crop enhancement by air anions. International Journal of Biometeorology. 1981;25: 309–321.
8. Morgan K. "Electroculture" gardening is trending. But does it work? The Washington Post. 30 Aug 2024.
9. Fromm J, Fei H. Electrical signaling and gas exchange in maize plants of drying soil. Plant Science. 1998;132: 203–213. doi:10.1016/S0168-9452(98)00010-7

10. Sukhov V, Gaspirovich V, Mysyagin S, Vodeneev V. High-Temperature Tolerance of Photosynthesis Can Be Linked to Local Electrical Responses in Leaves of Pea. Front Physiol. 2017;8: 763. doi:10.3389/fphys.2017.00763

11. Fromm J, Eschrich W. Electric Signals Released from Roots of Willow (Salix viminalis L.) Change Transpiration and Photosynthesis. Journal of Plant Physiology. 1993;141: 673–680. doi:10.1016/S0176-1617(11)81573-7

12. Herde O, Peña- Cortés H, Fuss H, Willmitzer L, Fisahn J. Effects of mechanical wounding, current application and heat treatment on chlorophyll fluorescence and pigment composition in tomato plants. Physiologia Plantarum. 1999;105: 179–184. doi:10.1034/j.1399-3054.1999.105126.x

13. Koziolek C, Grams TEE, Schreiber U, Matyssek R, Fromm J. Transient knockout of photosynthesis mediated by electrical signals. New Phytologist. 2004;161: 715–722. doi:10.1111/j.1469-8137.2004.00985.x

14. Dannehl D. Effects of electricity on plant responses. Scientia Horticulturae. 2018;234: 382–392. doi:10.1016/j.scienta.2018.02.007

15. Lidon FC, Ramalho JC, Henriques FS. Copper Inhibition of Rice Photosynthesis. Journal of Plant Physiology. 1993;142: 12–17. doi:10.1016/S0176-1617(11)80100-8

16. Malhi SS, Karamanos RE. A review of copper fertilizer management for optimum yield and quality of crops in the Canadian Prairie provinces. Can J Plant Sci. 2006;86: 605–619. doi:10.4141/P05-148

17. Kumar R, Mehrotra NK, Nautiyal BD, Kumar P, Singh PK. Effect of copper on growth, yield and concentration of Fe, Mn, Zn and Cu in wheat plants (Triticum aestivum L.). Journal of Environmental Biology. 2009.

18. Krofta K, Pokorný J, Kudrna T, Ježek J, Pulkrábek J, Křivánek J, et al. The effect of application of copper fungicides on photosynthesis parameters and level of elementary copper in hops. Plant Soil Environ. 2012;58: 91–97. doi:10.17221/437/2011-PSE

19. Jin M - F., You M - X., Lan Q - Q., Cai L - Y., Lin M - Z. Effect of copper on the photosynthesis and growth of *Eichhornia crassipes*. Luo Z - B., editor. Plant Biol J. 2021;23: 777–784. doi:10.1111/plb.13281

20. Vinit-Dunand F, Epron D, Alaoui-Sossé B, Badot P-M. Effects of copper on growth and on photosynthesis of mature and expanding leaves in cucumber plants. Plant Science. 2002;163: 53–58. doi:10.1016/S0168-9452(02)00060-2

21. Saathoff AJ, Welles J. Gas exchange measurements in the unsteady state. Plant Cell & Environment. 2021;44: 3509–3523. doi:10.1111/pce.14178

22. Blackman VH. Field experiments in electro-culture. The Journal of Agricultural Science. 1924;14: 240–267.

23. Ward RG. The influence of electric currents on the growth of tomator plants. Acta Physiologiae Plantarum. 1996;18: 121–127.

24. Smith DB, Solano F, Woodruff LG, Cannon WF, Ellefsen KJ. Geochemical and mineralogical maps, with interpretation, for soils of the conterminous United States. United States Geological Survey; 2017.

25. Ballabio C, Pangos P, Lugato E, Huang J-H, Orgiazzi A, Jones A, et al. Copper distribution in European topsoils: An assessment based on LUCAS soil survey. Science of The Total Environment. 2018;636: 10.1016/j.scitotenv.2018.04.268.

Tables

Table 1. Model comparison results for repeated measures analysis of height, SPAD, A_{net}, and g_{sw} for each of the four species (*: marginally significant, **: statistically significant).

	**Mustard Greens**		**Kale**			**Beets**			**Turnips**	
**Height**	$\chi 2$	*p*	$\chi 2$	*p*		$\chi 2$	*p*		$\chi 2$	*p*
week*treatment	17.11	0.07	9.15	0.52		20.75	0.05	*	6.08	0.81
week+treatment	2.11	0.35	3.39	0.18		0.93	0.63		4.37	0.11
**SPAD**										
week*treatment	4.04	0.95	4.04	0.95		10.25	0.42		5.97	0.82
week+treatment	4.48	0.11	0.42	0.81		2.20	0.33		2.54	0.28
**A_{net}**										
week*treatment	2.36	0.97	9.96	0.27		**20.42**	**0.00**	**	8.90	0.18
week+treatment	0.46	0.79	5.02	0.08		**8.66**	**0.01**	**	2.84	0.24
**g_{sw}**										
week*treatment	0.44	1	13.97	0.27		13.06	0.11		11.66	0.17
week+treatment	2.63	0.27	**9.87**	**0.01**	**	4.18	0.12		3.27	0.20

Table 2. ANCOVA tables for the relationships between A_{net} and g_{sw} for each of mustard greens, kale, beets, and turnips.

	**Mustard Greens**		**Kale**	
	Fvalue	Pvalue	Fvalue	Pvalue
log(gsw)	431.81	<0.001	610.68	<0.001
treatment	0.224	0.799	0.065	0.937
log(gsw):treatment	1.774	0.174	0.556	0.575

	**Beets**		**Turnips**	
	Fvalue	Pvalue	Fvalue	Pvalue
log(gsw)	143.19	<0.001	294.57	<0.001
treatment	2.294	0.106	1.303	0.276
log(gsw):treatment	2.188	0.117	0.893	0.413

List of Figures

Figure 1. Effects of week and copper treatment on A_{net} of **A)** mustard greens, **B)** kale, **C)** beets, and **D)** turnips. Points show mean ± 1 SE. Letters denote significant differences within weeks when a treatment x interaction was significant.

Figure 2. Effects of week and copper treatment on g_{sw} of **A)** mustard greens, **B)** kale, **C)** beets, and **D)** turnips. Points show mean ± 1 SE. Letters denote significant differences within weeks when a treatment x interaction was significant.

Figure 3. Linear regressions between A_{net} and g_{sw}, representing water user efficiency (WUE), for each of **A)** mustard greens, **B)** kale, **C)** beets, and **D)** turnips.

Figure 4. Bar charts of aboveground biomass production for beets, turnips, mustard greens, and kale. Bars show mean +/- 1 SEM. Letters denote statistically different groups.

Figure 5. Bar charts of belowground biomass production for beets and turnips. Bars show mean +/- 1 SEM. Letters denote statistically different groups.

Figure 1

Click here to access/download;Figure;Fig1.pdf

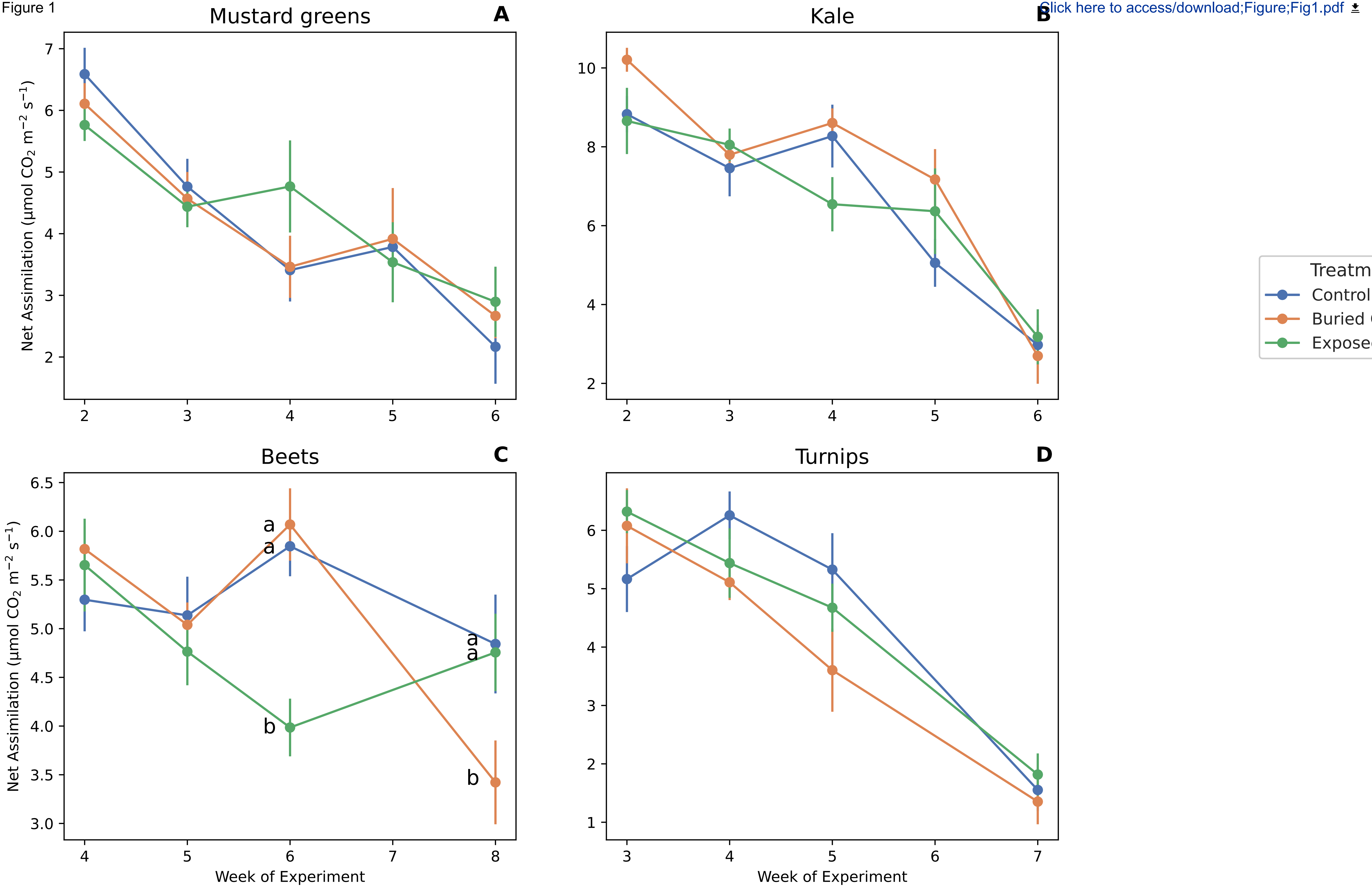

Figure 2

Click here to access/download;Figure;Fig2.pdf

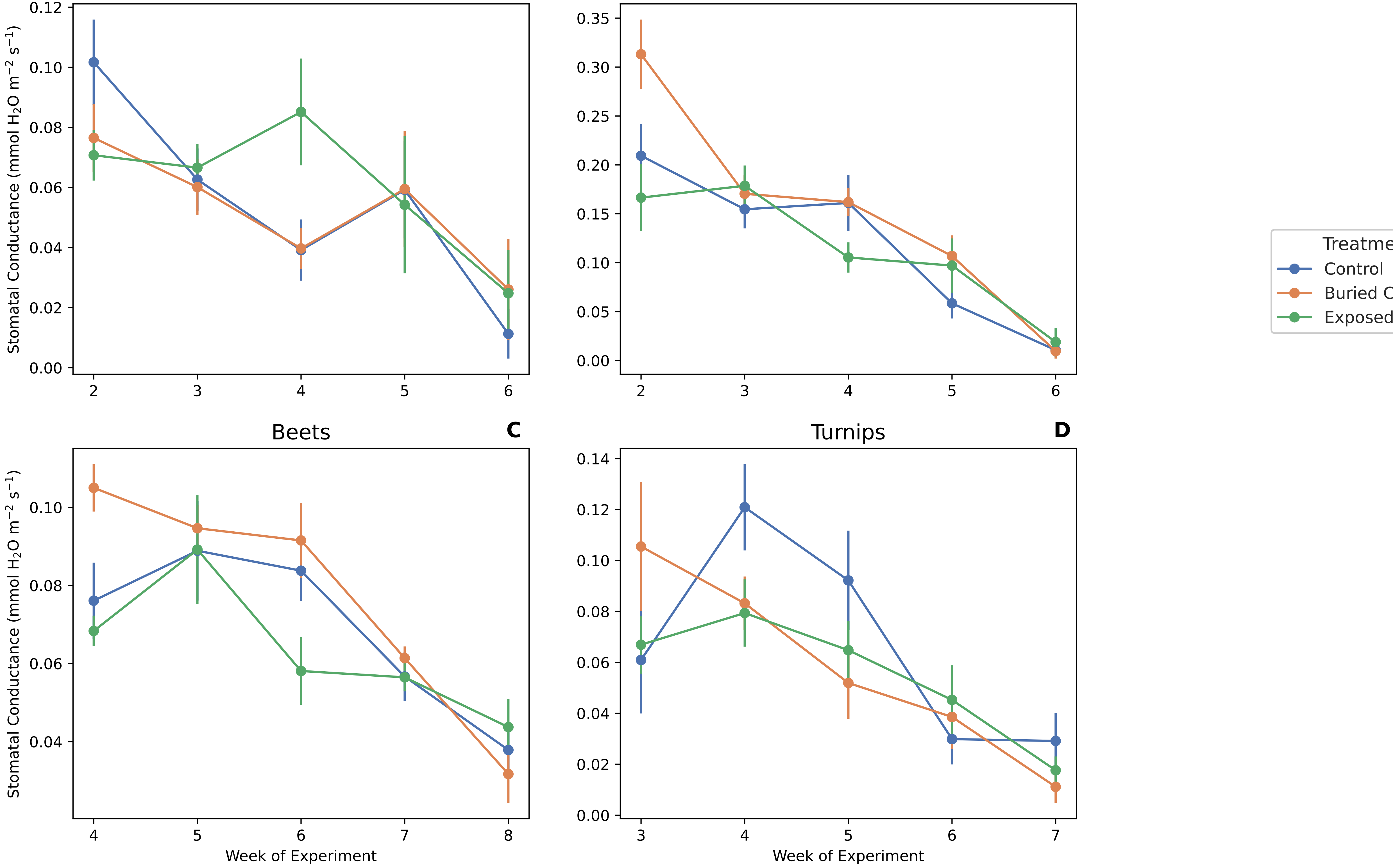

Figure 3

Click here to access/download;Figure;Fig3.pdf

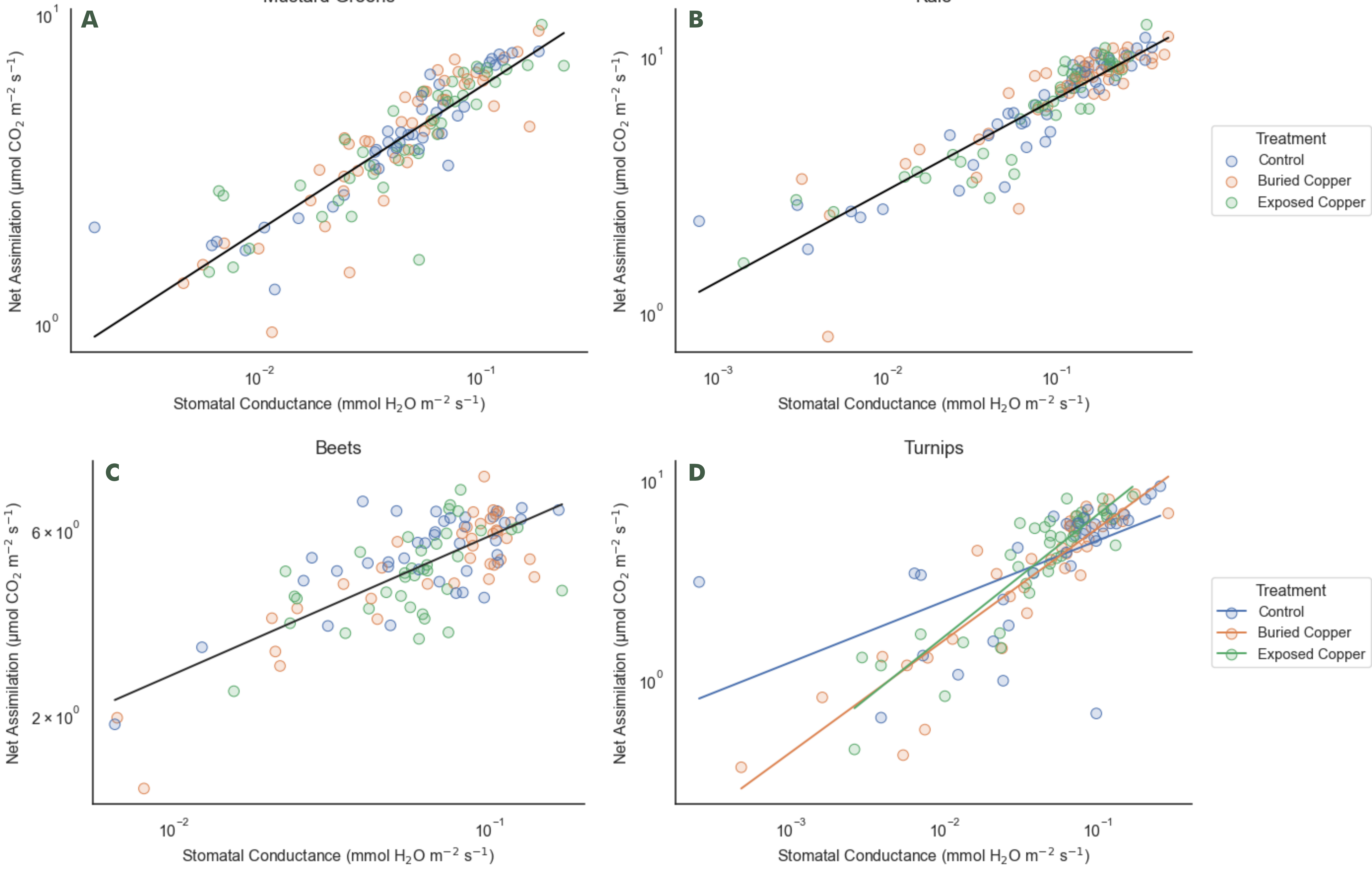

Figure 4

Click here to access/download;Figure;Fig4.pdf

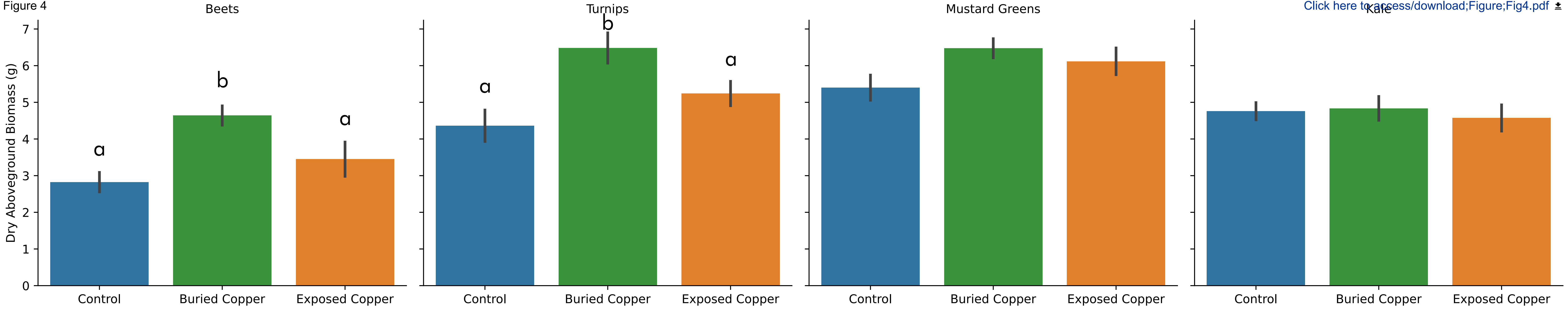

Figure 5

Click here to access/download;Figure;Fig5.pdf

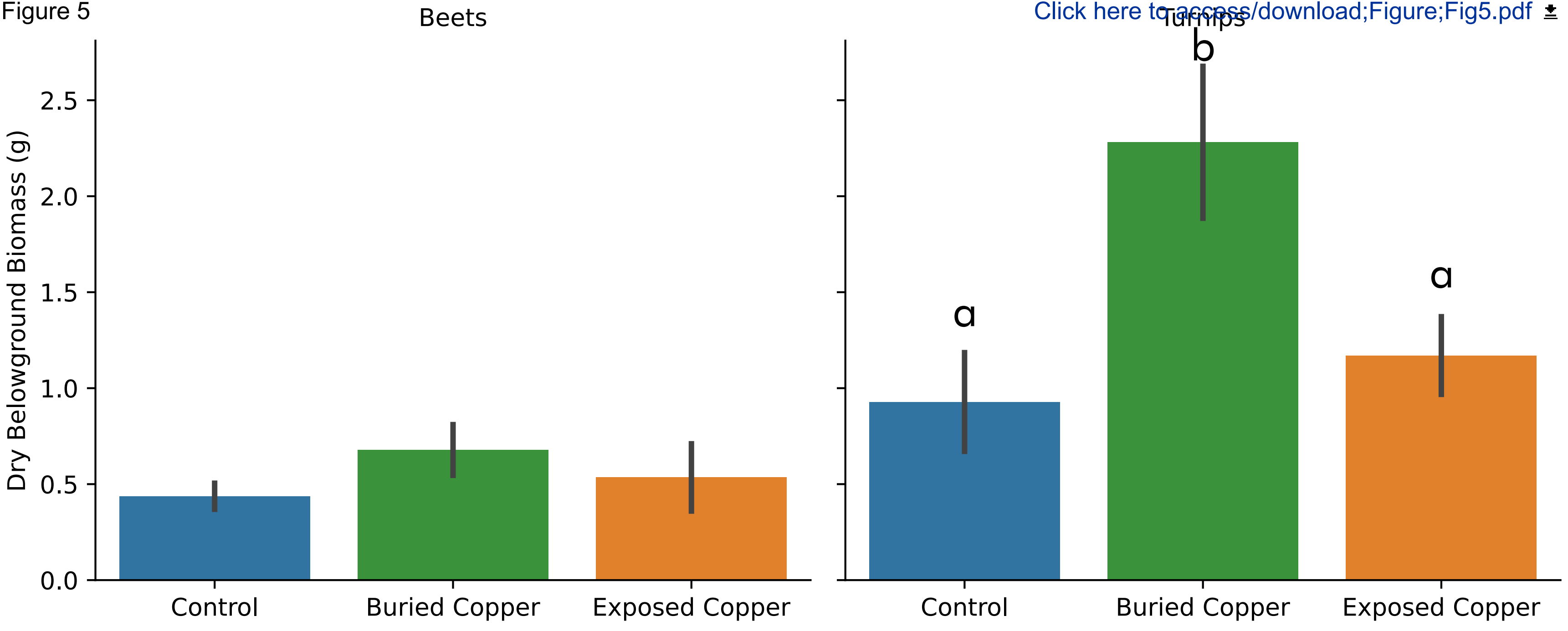

Supplement: Attachment — Submitted filename: PONE-D-25-18809.pdf [file pone.0329615.s001.pdf]
